# Piloting Psychology Annual Reviews as a Method of Measuring Psychological Distress and Quality of Life in Paediatric Renal Transplant Patients

**DOI:** 10.1155/2016/1685362

**Published:** 2016-11-14

**Authors:** Jade Bamford, Lucy Wirz

**Affiliations:** Great North Children's Hospital Kidney Team, Royal Victoria Infirmary, Newcastle upon Tyne Hospitals NHS Foundation Trust, Queen Victoria Road, Newcastle upon Tyne NE1 4LP, UK

## Abstract

Psychosocial distress and poorer quality of life after renal transplantation are common in children and young people. This has implications for medication adherence and survival. Posttransplant psychology annual reviews were introduced in one Paediatric Renal Service in the UK as a means of measuring psychological distress and quality of life, as well as facilitating identification of patients and parents/carers who would benefit from psychological intervention. The process of completing posttransplant psychology annual reviews is discussed within this paper. The posttransplant psychology annual review appointments identified patients experiencing depression and/or anxiety and problems in quality of life. These assessments have led to appropriate referrals to, and engagement with, the renal psychology service as well as with community tier 3 child and adolescent mental health services. The posttransplant psychology annual review will continue to be completed at this UK site and discussions will be undertaken with other paediatric renal transplant services to consider whether these could be introduced at a national level to facilitate collection of longitudinal data regarding long-term psychosocial impact of paediatric renal transplantation and its effect on quality of life.

## 1. Introduction

There has been a growing body of research into the psychosocial impact of renal transplantation and its impact on quality of life. Children and young people after renal transplantation report higher mental health difficulties and lower quality of life compared with their healthy peers [1]. Examples of the difficulties experienced by children and young people include depression [2, 3], generalised, social and health anxiety [3, 4], cognitive difficulties [3, 5], body image concerns [4, 6, 7], difficulties adjusting to a “healthy” status [4], sleep disturbances [4], and pain [3]. All of these difficulties can make it harder for young people to attend school and spend time with peers [8] thus further impacting on quality of life.

Greater levels of psychological distress have been found to be correlated with poorer medication adherence after transplantation [9]. This suggests that psychological well-being can have a direct impact on the longer-term outcome of a transplant. Therefore maintaining and promoting positive psychological well-being also has positive implications for medical outcomes.

Risk factors associated with psychological distress after renal transplantation include shorter time since transplant; younger age at time of transplantation; congenital disease; existing psychological diagnosis; neurological disease; low sociodemographic status; family conflict; parental factors, for example, parental anxiety, psychosocial distress, difficulties in parents' physical functioning, or lower parental quality of life; and individual/transplant factors, for example, adherence, frequency of rescheduled appointments, and presence of a transplant rejection episode [8, 10].

In order to provide holistic care to paediatric renal transplant patients, it is important to identify those who are experiencing psychological distress. Within the Paediatric Renal Service at this UK site, pretransplant assessments of psychosocial well-being are carried out routinely for all renal transplant patients. Pretransplant assessment is an opportunity to identify the areas of strengths and needs for a patient and their family and offer timely intervention for those experiencing psychological distress. Having an increased awareness of the risk factors for psychological distress assists but does not ensure reliable prediction of patients who will experience psychological difficulties after transplant [9]. An assessment of psychosocial well-being after transplant is, therefore, recommended to identify signs of psychological distress [4, 9].

The Paediatric Renal Service at this UK site introduced psychology annual review appointments for all posttransplant patients as a method of screening for psychosocial issues. The aim of the psychology annual review appointment is to facilitate a more holistic model of care by identifying areas of difficulties for patients and families to enable psychologists to provide timely and targeted intervention to improve quality of life and reduce symptoms of psychological distress.

This paper outlines and evaluates the process of introducing psychology annual reviews for paediatric renal transplant patients at one UK site. It aims to investigate whether psychology annual reviews have facilitated identification of patients and families experiencing psychosocial difficulties. Reported psycho-social difficulties were compared with accessing of psychological services (e.g. renal psychology service, Child and Adolescent Mental Health Services) to assess whether the psychology annual reviews were predictive of need for psychological input.

## 2. Materials and Methods

### 2.1. Procedure

A psychology annual review appointment was offered to all patients with a kidney transplant engaged in the Paediatric Renal Service at one UK site and this formed part of the medical transplant annual review. Medical transplant annual reviews are completed one year after kidney transplant and then each consecutive year until the patient transitions to adult renal services, usually aged 17-18 years. 31 patients were eligible to complete the psychology annual review between January and December 2014.

The psychology annual review was conducted by a supervised assistant psychologist and consisted of a combination of clinical interview and psychometric measures. Firstly, the assistant psychologist discussed the psychology annual review process with the parent and/or patient and requested completion of a consent form. The assistant psychologist gave instructions on how to complete all psychometric measures. Most psychometric measures were completed in a clinic room supervised by the assistant psychologist. The assistant psychologist reviewed the psychometric measures with the parents and/or patient and conducted a clinical interview. Several patients and parents/carers did not have time to complete the consent form and measures during the appointment and took them home. All measures taken home were accompanied by a pre-paid stamped addressed envelope to return them in the post once completed. Following completion of the clinical interview and psychometric measures, a letter was written to patients/families to provide an outline of the discussion and any key issues identified. With parental consent, this letter was copied to the medical and nursing team for the purpose of information sharing. Previous involvement with psychological services and/or involvement with Child and Adolescent Mental Health Services (CAMHS) was recorded for all consented patients.

### 2.2. Psychometric Measures

Psychology annual review appointments utilised a battery of six psychometric measures to screen for psychosocial concerns. These measures were selected because they have been developed and validated for measuring patient and carer psychological well-being and quality of life in the context of physical health problems [11–15].

The measures completed by patients and/or parents/carers are detailed below (see Table 1 to identify questionnaires administered dependent on age of patient).

#### 2.2.1. Paediatric Quality of Life Generic Core Scales: Parent Version (Peds-QL Version 4.0) [12, 16]

The Peds-QL measures parents'/carers' feelings about their child's quality of life. There are specific questionnaires for 0–12 months and 13–24 months. It provides information about physical functioning, physical symptoms, emotional functioning, social functioning and cognitive functioning.

A 5-point response scale is utilised (0 = never a problem; 1 = almost never a problem; 2 = sometimes a problem; 3 = often a problem; 4 = almost always a problem).

#### 2.2.2. Paediatric Quality of Life Transplant Module: Parent Version (Peds-QL Version 4.0) [12, 16]

The Peds-QL measures parents'/carers' feelings about their child's health related quality of life following a transplant. There are specific questionnaires for parents/carers of children aged 5–7 years, 8–12 years, and 13–18 years. It provides information about barriers to medical regimen adherence, medication side effects, social relationships and transplant, physical discomfort, worries related to health status, treatment anxiety, impact of transplant on appearance and communication with medical staff and others regarding transplant issues.

A 5-point response scale is utilised (0 = never a problem; 1 = almost never a problem; 2 = sometimes a problem; 3 = often a problem; 4 = almost always a problem).

#### 2.2.3. Paediatric Quality of Life Transplant Module: Patient Version (Peds-QL Version 4.0) [12, 16]

The Peds-QL measures patients' feelings about their health related quality of life following a transplant. There are specific questionnaires for patients aged 5–7 years, 8–12 years, and 13–18 years. It provides information about barriers to medical regimen adherence, medication side effects, social relationships and transplant, physical discomfort, worries related to health status, treatment anxiety, impact of transplant on appearance, and communication with medical staff and others regarding transplant issues.

For the questionnaires for 5–7 years a 3-point response scale is utilised (0 = not at all a problem; 2 = sometimes a problem; 4 = almost always a problem). For the questionnaires for 8–12 years and 13–18 years, a 5-point response scale is utilised (0 = never a problem; 1 = almost never a problem; 2 = sometimes a problem; 3 = often a problem; 4 = almost always a problem).

#### 2.2.4. Paediatric Inventory for Parents (PIP) [13]

The PIP is a parent report questionnaire, measuring the frequency and difficulty of different events which parents/carers of children who have a serious illness sometimes face. The questionnaire looks at four domains: communication, medical care, emotional distress, and role function.

Parents are asked to rate how frequent and how difficult each event is using a 5-point response scale (frequency: 1 = never, 2 = rarely, 3 = sometimes, 4 = often, and 5 = very often; difficulty: 1 = not at all, 2 = a little, 3 = somewhat, 4 = very much, and 5 = extremely).

#### 2.2.5. Paediatric Index of Emotional Distress (PI-ED) [17]

The PI-ED is a questionnaire completed by patients (8–15 years) to assess if they are experiencing symptoms of emotional distress. The questionnaire is designed to be used with children with physical health problems that is, in paediatric clinics and hospitals, but can also be used with the general population. Patients are asked to rate the frequency of positive and negative feelings and emotions.

A 4-point response scale is utilised (always, a lot of the time, sometimes, and not at all).

#### 2.2.6. Hospital Anxiety and Depression Scale (HADS) [18]

The HADS is completed by patients (16–18 years) to assess if they are experiencing symptoms of anxiety and depression. The questionnaire is designed to be used with adults with physical health problems that is, in clinics and hospitals but can also be used with the general population. The measure also provides an indication of symptom severity.

A 4-point response scale is utilised (e.g., most of the time; a lot of times; from time to time; not at all).

### 2.3. Clinical Interview

Following completion of psychometric measures, the assistant psychologist reviewed the responses with the parents and/or patients; this formed part of the clinical interview. The assistant psychologist raised with the parent and/or patient any responses suggesting clinical concern. Clinical concern and further exploration was deemed to be required in the following circumstances.

#### 2.3.1. Peds-QL Transplant Parent and Patient Version

Any individual questions in the Peds-QL which received a score of 2 (sometimes a problem), 3 (often a problem) or 4 (almost always a problem) were further explored with the responder (parent or patient) to assess for psychological distress or negative impact on quality of life.

#### 2.3.2. PIP

Any individual questions in the PIP which received a score of 3 (sometimes), 4 (often) or 5 (very often) for frequency of events and/or which received a score of 3 (somewhat), 4 (very much) or 5 (extremely) for difficulty of events were further explored with the parent. This provided a forum for parents to discuss the challenges presented to themselves as carers for a child or young person with a physical illness.

#### 2.3.3. PI-ED

Total scores above the level of clinical significance (10 for boys, 11 for girls) were explored further with the patient and discussed with the parents providing the opportunity to assess the mood of the child/young person.

#### 2.3.4. HADS

Total scores above the level of clinical significance (8) for anxiety and/or depression were explored further with the patient and discussed with the parents providing the opportunity to assess the mood of the young person.

Supplementary information was gathered from the parent and/or patient during the clinical interview. Topics explored included challenging behaviour and conduct difficulties; sleep difficulties; toileting; obsessive and compulsive behaviours; eating and feeding; peer relationships and social skills; confidence and self-esteem; and impact on parents, siblings, and other family members.

The combination of psychometric measures and supplementary information allowed the assistant psychologist, under the supervision of a consultant clinical psychologist, to determine whether or not a patient and/or family required ongoing psychological support. This has been termed the “psychology outcome.” There were 5 possible psychology outcomes following the completion of the psychology annual reviews:

1. Patient already seeing renal psychologist: patients with this outcome are actively seeing an assistant psychologist or clinical psychologist within the Paediatric Renal Service.
2. Patient referred to renal psychology: patients with this outcome are newly referred to the psychology service within the Paediatric Renal Service as a result of their psychology annual review appointment and have opted in to an appointment with an assistant psychologist or clinical psychologist.
3. Patient referred to renal psychology but did not engage: patients with this outcome are newly referred to the psychology service within the Paediatric Renal Service as a result of their psychology annual review appointment but have opted out of an appointment with an assistant psychologist or clinical psychologist.
4. Patient referred to CAMHS: patients with this outcome are newly referred to CAMHS following an identified complex mental health need that is not related to their kidney disease and/or kidney transplant, for example, assessments for Autism Spectrum Disorder or Obsessive Compulsive Disorder. Alternatively, due to the nature of the service providing regional care, some parents/patients may request a referral to CAMHS, rather than the renal psychology service, if the distance of travel between home and the regional hospital base is felt to be too burdensome.
5. No further action/referral needed: patients with this outcome were identified to not require any ongoing psychological support or assessment at the current time. They may have received support from the psychology service within the Paediatric Renal Service or from CAMHS previously but are not actively receiving support at present.

## 3. Results

### 3.1. Demographics

In 2014, there were 31 transplant patients in the Paediatric Renal Service at this UK site. Psychology annual reviews were conducted with 20 (64.5%; 9 male, 11 female) patients and/or parents/carers. 11 patients and parents/carers did not complete the psychology annual review because questionnaires were taken home by families and not returned (4); patient did not attend their scheduled annual review appointment in 2014 (2); no psychologist was able to be present in clinic during the patient's annual review appointment (2); and patients transitioned to adult renal services before their annual review appointment in 2014 (3). The age of patients who completed, or whose family completed, questionnaires ranged from 3 to 17 years (mean = 13.4 years) and number of years post-transplant ranged from 1 to 13 years (mean = 6.1 years).

As summarised in Table 1, the psychometric measures administered depended upon patient age.  Table 2 outlines how many questionnaires of each type were completed by patients and by parents/carers.  Table 3 outlines patients' psychological involvement prior to the psychology annual review appointment in 2014.

### 3.2. Psychology Outcome

Table 4 demonstrates that as a result of the psychology annual review appointment, three patients have been newly referred to renal psychology and two patients have been newly referred to CAMHS. A further two patients were identified to have ongoing psychological need but the patient and their family chose to not engage with renal psychology services.

Presenting psychological needs which led to referral to renal psychology services included: patient adjustment to their health condition, child behaviour issues, procedural anxiety and self-harm. Patients were referred to CAMHS if they presented with complex mental health needs not specifically related to their physical health condition and/or at parental request when the families geographical location did not enable them to routinely access the regional hospital base.

### 3.3. Measures

#### 3.3.1. Parent Questionnaire: Paediatric Quality of Life Generic Core Scales (Peds-QL)

No patient included in this study was aged 2 years or below and therefore the Peds-QL Generic Core Scales was not administered and shall not be discussed further in the results or discussion. However, it is helpful moving forward to highlight the methodology for assessing younger children using the psychology annual review process.

#### 3.3.2. Parent Questionnaire: Paediatric Quality of Life Transplant Module (Peds-QL)

**Figure 1:**
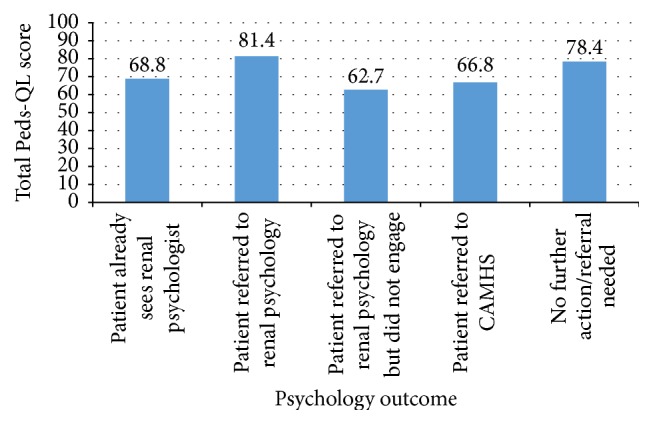
Mean total scaled score, as rated by parents/carers, for each psychological outcome.

Scores on individual items on the Peds-QL are converted into a total scaled score ranging from 0 to 100. Higher scores indicate fewer problems with quality of life. Total scaled scores as rated by parents, ranged from 40.2 to 96.2 (mean = 73.9).  Figure 1 displays the mean total scaled score for each psychological outcome.

Figure 1 suggests that lower scores on the parent report Peds-QL is not predictive of need for psychological intervention.

#### 3.3.3. Patient Questionnaire: Paediatric Quality of Life Transplant Module (Peds-QL)

**Figure 2:**
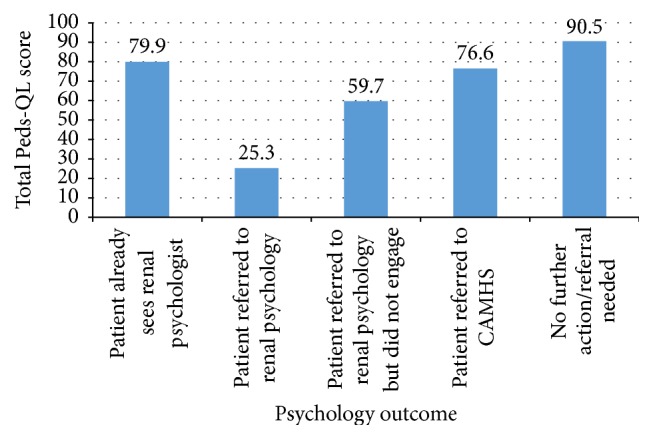
Mean total scaled score, as rated by patients, for each psychological outcome.

Scores on individual items are converted into a total scaled score ranging from 0 to 100. Higher scores indicate fewer problems. Total scaled scores as rated by patients, ranged from 25.3 to 96.6 (mean = 79.6).  Figure 2 displays the mean total scaled score for each psychological outcome.

Figure 2 suggests that patients that did not require further psychological intervention have the highest quality of life, in the sample of 17 patients that completed the questionnaire. Patients who were already accessing renal psychology services had higher quality of life relative to those who were newly referred to renal psychology services. Patients referred to CAMHS reported higher quality of life on the Peds-QL relative to patients referred to renal psychology service, suggesting that patients referred to CAMHS were experiencing mental health difficulties not directly related to their physical health condition. The patient rated Peds-QL therefore appears to be more predictive of need for psychological intervention and can distinguish between patients with psycho-social difficulties specific to their health condition and patients with generic mental health difficulties.

**Figure 3:**
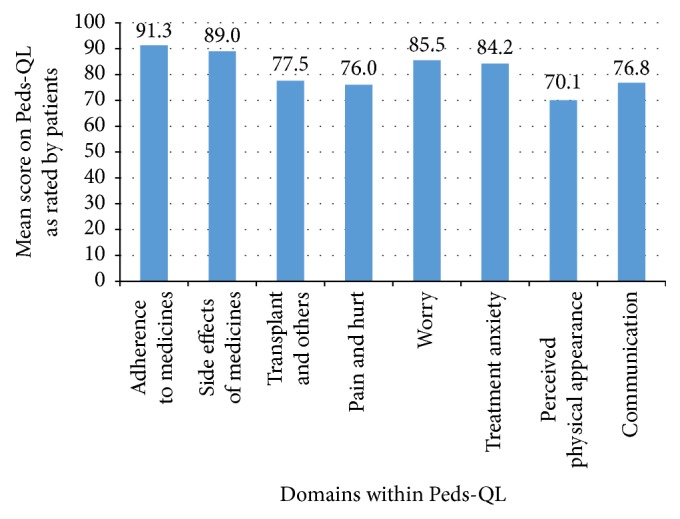
Mean score on Peds-QL as rated by patients for each of the 8 domain scores.

Figure 3 demonstrates that the greatest areas of concern for children and young people in relation to their transplant are their perceived physical appearance as a result of transplantation; physical discomfort, pain and hurt; communication with medical staff and others regarding transplant issues; and how their transplant affects their social relationships with others. Figure 3 suggests that the areas of least concern for children and young people were adherence to medication; side effects of medication; worry about future ill-health; and treatment anxiety.

#### 3.3.4. Paediatric Index of Emotional Distress (PI-ED)

Each response on the PI-ED has a corresponding score between 0 and 3. All scores are summed together to get a total distress score. Higher scores indicate greater levels of emotional distress. Elevated emotional distress (clinical caseness) is 11 and over for girls and 10 and over for boys. 11 patients completed the PI-ED.

**Figure 4:**
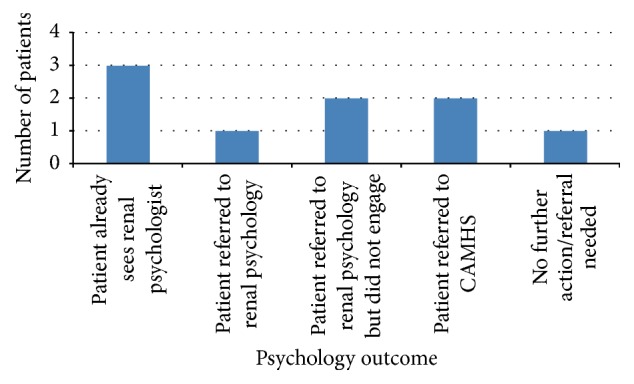
Psychology outcomes for patients in the clinically significant range for symptoms of emotional distress as reported on the PI-ED.

Nine patients (82%) who completed the PI-ED scored within the clinically significant range for symptoms of anxiety and/or depression.  Figure 4 summarises the psychology outcome for these nine patients who scored within the clinically significant range.  Figure 4 shows that 66.6% of patients who reported that they were experiencing clinically significant levels of emotional distress were already receiving support from the renal psychologists or were newly referred to psychology services in the renal team or in CAMHS. Two patients (22.2%) were referred to psychology services but did not engage. Only 1 patient (11.1%) scoring clinically significant for symptoms of emotional distress did not require any psychological follow-up or intervention as determined by their clinical interview. In this case, patient's responses on the PI-ED may have been elevated as a result of other factors such as sleep difficulties or physical symptoms.

#### 3.3.5. Hospital Anxiety and Depression Scale (HADS)

Responses to each question in the HADS has a corresponding a score between 0 and 3. Scores are summed together to get an overall anxiety symptom score and an overall depression symptom score. Total symptom scores indicate the severity of anxiety and depression (0–7 = normal; 8–11 = mild; 12–14 = moderate; 15–21 = severe).

Six patients completed the HADS. All six patients (100%) scored within the normal range for symptoms of depression and four patients (67%) within the normal range for anxiety symptoms. Two patients (33%) reported experiencing mild symptoms of anxiety, but neither required any psychology follow-up or intervention following further exploration during the clinical interview. Scores between 8 and 10 are in the borderline range and thus not decisively indicative of mood disorder [19]. Therefore, our findings that mild anxiety did not require psychological intervention are consistent with other studies.

#### 3.3.6. Paediatric Inventory for Parents (PIP)

Scores for each event are totaled together to give a total frequency and total difficulty score. Higher scores indicate greater frequency and greater perceived difficulty of events. Scores can range from 42 to 210. Total frequency scores ranged from 45 to 138 (mean = 78.5). Total difficulty scores ranged from 44 to 112 (mean = 69.4). This highlights that all parents were experiencing some events (i.e. reported events occur at least “rarely”) related to their child's medical condition and that all of them found this to be at least a little difficult (i.e. rated events to be above “not at all difficult”). The difficulty of events relating to their child's medical condition was further explored during the clinical interview. Parents were signposted to relevant agencies as required to support them with their personal well-being, for example, signposting to GP, Paediatric Renal Service social worker or adult mental health practitioner.

## 4. Discussion

Posttransplant psychology annual review appointments have been successfully introduced within the Paediatric Renal Transplant Service as a routine element of the posttransplant annual review process. Studies have previously demonstrated that there is stigma attached with acknowledging psychological difficulties [20–22]. Familiarity with the psychologists and the role of psychologists through routine psychology appointments at annual review may help to reduce this stigma within the patient/parent group, enabling psychosocial concerns to be raised more openly and targeted psychological intervention to be provided. However, the time that is required to complete the psychology element of the annual review could be perceived as an additional demand by some families. It would be beneficial, in the future, to gather feedback from patients and parents/carers about the acceptability of the psychology annual review process including the appropriateness of questionnaires.

The questionnaires, in combination with clinical interviews, used in the psychology annual review appointment have helped to identify patients experiencing psychological distress and problems in quality of life. This has increased opportunities to offer advice and psychological support, including engaging new patients with the renal psychology service, and new referrals to CAMHS. These patients have previously not presented overtly with concerns when seen by the medical and nursing team and therefore identifying psycho-social issues that have been overlooked on other occasions is a positive outcome. For those patients who were already engaged with the renal psychology service, the psychology annual review has acted as an opportunity to evaluate patient progress. Some patients were identified as having psychological need during their annual review but did not wish to engage in psychology support at this time. Identifying this group of patients allowed all members of the multidisciplinary renal team to be mindful of potential psychological risk and to rediscuss issues at subsequent clinic appointments, where appropriate. Of those patients who did not require further intervention following the psychology annual review appointment, two thirds have previously seen a psychologist for psycho-social issues related to their transplant and thus having a mechanism for yearly follow-ups with this patient group is beneficial.

Incorporating the psychology annual review into the medical posttransplant annual review has increased recognition within the team of the psychosocial impact of kidney transplantation. Copying letters to the medical and nursing staff to outline key issues presented by patients has been valuable information sharing to support patients. On the Peds-QL, patients frequently reported difficulties communicating with doctors and nurses. Therefore, young people may not always feel able to raise issues relating to their transplant or psychological well-being with a medical or nursing member of the team. For this reason, it could be helpful to have the psychology annual review appointment as an alternative forum for these discussions. Delivering more psychoeducation and training to the medical/nursing team about the psychosocial impact of renal transplantation may also be helpful in further promoting appropriate discussion and engagement with young people.

There was some consistency between the main issues reported by patients on the Peds-QL and the literature [4, 6, 7], for example, physical discomfort and physical appearance. Having the mechanism to identify these issues has facilitated appropriate and timely psychological intervention. The areas on the Peds-QL in which patients reported fewest concerns (adherence to medication, side effects of medication, worry about future ill-health, and treatment anxiety) are most frequently monitored by the medical team due to the immediate impact on a young person's physical health. It is therefore not surprising that there were fewest concerns reported in these areas. A young person with treatment anxiety, for example, would have an immediate impact on their receipt of medical intervention and therefore it is likely that a referral would have already have been made to the renal psychologists for support regarding these issues. Previous studies have identified that patients over-estimate treatment adherence [23] and therefore, on the Peds-QL, patients may have chosen to not acknowledge that there is a problem with adherence. In the adult transplant population, disclosures regarding medication adherence were found to be more accurate when this was disclosed to an independent researcher, rather than to clinical staff [24]. This suggests that there may be a benefit of having a non-medical member of the team to also ask about medication adherence.

The literature highlighted that paediatric kidney transplant patients are at an increased risk of experiencing symptoms of depression and anxiety [2–4]. During the psychology annual review appointment, 11 (64%) patients who completed either the PI-ED or the HADS scored within the clinical range for symptoms of depression and/or anxiety. Of these 11 patients, only three (28%) did not require ongoing psychological support when this was further explored during the clinical interview. This suggests that the PI-ED and the HADS, accompanied by the clinical interview, were effective in identifying low mood and anxiety in this patient population. It also underlines the significance of psychological services within Paediatric Renal Services nationally, and the pertinence of enquiring actively about more internalising psychological difficulties such as anxiety and low mood. Often these difficulties are less readily highlighted by children and young people, and are usually less obvious to others including parents/carers, schools and medical teams, compared to more externalising difficulties such as aggressive or oppositional behaviour.

The questionnaires used within the psychology annual reviews did not measure all of the problem areas reported when clinical interviews were conducted with young people and their families. For example, a common difficulty reported by parents during the clinical interview was behavioural issues. None of the questionnaires administered account for behaviour and thus it could be helpful to consider incorporating an additional questionnaire such as the Strengths and Difficulties Questionnaire (SDQ) [25], which assesses emotional-behavioural difficulties more generally. Incorporating this questionnaire would increase the demands placed on parents and may affect the acceptability and engagement with the annual review process and therefore should be considered carefully. Patterns of referrals to the paediatric renal psychology service suggest that parents/carers and the medical team are more able to recognise where input is needed around externalising issues such as challenging behaviour.

Parental well-being in the context of having a child with health issues was assessed using the PIP. All parents reported that they experienced events which they found difficult in relation to their child's physical health condition. Parents have been signposted, as appropriate, to GP, adult mental health services, benefits advisers and to the Paediatric Renal Service social worker as a result of responses on psychometric measures and information collected during clinical interview. Details of signposting and engagement with services have not been recorded as part of this study and so cannot be reported at the present time. Moving forward, it would be useful to record advice and signposting given to parents during the psychology annual review appointment with the research data to monitor informal psychological support provided. It is also important to continue to collect information regarding parental well-being as this has been identified as a risk factor for psychological distress post-kidney transplantation [8, 10].

The administration of questionnaires and conducting clinical interviews requires the use of psychology staff resource which is limited within paediatric renal psychology services nationally. The psychometric measures provide an indication of patients who are experiencing emotional distress and lower quality of life. However, administration of the measures alone does not reliably identify patients who do and do not require ongoing psychological support or which service would be best placed to provide that support. The PI-ED and the HADS are designed to be suggestive of mood and anxiety disorders and therefore require further exploration by an experienced clinician. Currently, there are no established cut-off points for clinical significance on the Peds-QL Transplant and the PIP. Further collection of Peds-QL and PIP data in the paediatric renal transplant population may facilitate the development of normative data for this population which would provide an indication for when scores are elevated and therefore when patients/parents are experiencing greater levels of distress. This may help in identifying patients requiring psychological input; however, the incorporation of the clinical interview to gain additional qualitative information from families is invaluable.

## 5. Conclusion

The introduction of psychology annual reviews has enhanced the quality of care provided to patients and families accessing the Paediatric Renal Service at this UK site. The posttransplant psychology annual review appointments have enabled patients and families who are experiencing psychosocial difficulties to be identified and supported. Referrals to the renal psychology service and to CAMHS have been made as a direct result of the questionnaires and clinical interview administered at psychology annual review appointments.

The conclusions which can be drawn from this study at the present time are somewhat limited due to the small sample size (sample size = 20) and collection of data from 2014 only. Continuing to collect this data over an extended time-frame would provide longitudinal information about the difficulties young people face post-kidney transplant and provide an indication of whether psychological interventions have improved psychological well-being and quality of life. The authors have begun discussions with psychologists working within paediatric renal transplant services across the UK to consider whether post-transplant annual reviews could be introduced at a national level. This would facilitate collection of longitudinal data from a larger sample regarding long-term psychosocial impact of paediatric renal transplantation and its effect on quality of life.

## Figures and Tables

**Table 1:** Overview of psychometric measures administered to patients and/or parents/carers depending upon patient age.

Age of patient	Parent/carer to complete			Patient to complete		
	Peds-QL Generic Core Scales	Peds-QL Transplant Module	PIP	Peds-QL Transplant Module	PI-ED	HADS
0–2 years	√		√			
2–4 years		√	√			
5–7 years		√	√	√		
8–12 years		√	√	√	√	
13–15 years		√	√	√	√	
16–18 years		√	√	√		√

**Table 2:** Number of completed questionnaires and percentage completed from those eligible within the sample that completed a psychology annual review.

Questionnaires	Number of questionnaires completed by parents/patients (percentage of parents/patients completed from eligible age range)
Peds-QL Generic Core Scales, parent-rated	0 (100%)
Peds-QL Transplant Module, parent-rated	16 (80%)
Paediatric Inventory for Parents	12 (60%)
Peds-QL Transplant Module, patient-rated	17 (100%)
Paediatric Index of Emotional Distress	11 (100% + 2 patients aged 16 years)
Hospital Anxiety and Depression Scale	6 (75% remaining, 25% completed PI-ED)

**Table 3:** Psychological involvement before patients' 2014 psychology annual review appointment.

Psychology involvement before psychology annual review	Number of patients (out of 20^*∗*^)
Patient already sees renal psychologist	4
Patient already sees CAMHS	0
Patient has previously seen renal psychologist but is now discharged	12
Patient has previously seen CAMHS but is now discharged	3
No previous psychological intervention needed	4

^*∗*^Note: 2 patients have previously seen renal psychologist and CAMHS but are now discharged from both services. 1 patient was seeing renal psychologist at time of review and has previously seen CAMHS but is now discharged from CAMHS.

**Table 4:** Psychological involvement following completion of patients' 2014 psychology annual review appointment.

Psychology outcome as a result of psychology annual review	Number of patients (out of 20)
Patient already sees renal psychologist	4
Patient referred to renal psychology	3
Patient referred to renal psychology but did not engage	2
Patient referred to CAMHS	2
No further action/referral needed	9

## References

[1] Diseth T. H., Tangeraas T., Reinfjell T., Bjerre A. (2011). Kidney transplantation in childhood: mental health and quality of life of children and caregivers. *Pediatric Nephrology*.

[2] Karaminia R., Tavallaii S. A., Lorgard-Dezfuli-Nejad M. (2007). Anxiety and depression: a comparison between renal transplant recipients and hemodialysis patients. *Transplantation Proceedings*.

[3] Tong A., Tjaden L., Howard K., Wong G., Morton R., Craig J. C. (2011). Quality of life of adolescent kidney transplant recipients. *The Journal of Pediatrics*.

[4] Ranawaka P. R. D., Abeysekera C. K., Gamage M. P. (2015). Psychosocial outcomes of children and parents after renal transplantation. *Sri Lanka Journalof Child Health*.

[5] Haavisto A., Korkman M., Holmberg C., Jalanko H., Qvist E. (2012). Neuropsychological profile of children with kidney transplants. *Nephrology Dialysis Transplantation*.

[6] Morel P., Almond P. S., Matas A. J. (1999). Long-term quality of life after kidney transplantation in childhood. *Transplantation*.

[7] Krmar R. T., Eymann A., Ramirez J. A., Ferraris J. R. (1997). Quality of life after kidney transplantation in children. *Transplantation*.

[8] Haavisto A., Korkman M., Sintonen H. (2013). Risk factors for impaired quality of life and psychosocial adjustment after pediatric heart, kidney, and liver transplantation. *Pediatric Transplantation*.

[9] Dew M. A., Dabbs A. D., Myaskovsky L. (2009). Meta-analysis of medical regimen adherence outcomes in pediatric solid organ transplantation. *Transplantation*.

[10] Devine K. A., Reed-Knight B., Loiselle K. A., Simons L. E., Mee L. L., Blount R. L. (2011). Predictors of long-term health-related quality of life in adolescent solid organ transplant recipients. *Journal of Pediatric Psychology*.

[11] Varni J., Seid M., Kurtin P. (2001). PedsQL 4.0: reliability and validity of the paediatric quality of life inventory version 4.0 generic scales in health and patients populations. *Medical Care*.

[12] Weissberg-Benchell J., Zielinski T. E., Rodgers S. (2010). Pediatric health-related quality of life: feasibility, reliability and validity of the PedsQL™ transplant module. *American Journal of Transplantation*.

[13] Streisand R., Braniecki S., Tercyak K. P., Kazak A. E. (2001). Childhood illness-related parenting stress: the pediatric inventory for parents. *Journal of Pediatric Psychology*.

[14] O'Connor S., Ferguson E., Carney T., House E., O'Connor R. C. (2016). The development and evaluation of the Paediatric Index of Emotional Distress (PI-ED). *Social Psychiatry and Psychiatric Epidemiology*.

[15] Bjelland I., Dahl A. A., Haug T. T., Neckelmann D. (2002). The validity of the Hospital Anxiety and Depression Scale. An updated literature review. *Journal of Psychosomatic Research*.

[16] Varni J. W., Seid M., Rode C. A. (1999). The PedsQL™: measurement model for the pediatric quality of life inventory. *Medical Care*.

[17] O'Connor S., Carney T., House E., Ferguson E., Caldwell F., O'Connor R. (2010). Revision of the Hospital Anxiety and Depression Scale (HADS) to produce the Paediatric Index of Emotional Distress (PI-ED). *Patient Reported Outcomes Newsletter*.

[18] Zigmond A. S., Snaith R. P. (1983). The hospital anxiety and depression scale. *Acta Psychiatrica Scandinavica*.

[19] Snaith R. P. (2003). The hospital anxiety and depression scale. *Health and Quality of Life Outcomes*.

[20] Crisp A. H., Gelder M. G., Rix S., Meltzer H. I., Rowlands O. J. (2000). Stigmatisation of people with mental illnesses. *British Journal of Psychiatry*.

[21] Reavley N. J., Jorm A. F. (2011). Recognition of mental disorders and beliefs about treatment and outcome: findings from an Australian national survey of mental health literacy and stigma. *Australian and New Zealand Journal of Psychiatry*.

[22] Wang J., Lai D. (2008). The relationship between mental health literacy, personal contacts and personal stigma against depression. *Journal of Affective Disorders*.

[23] Waterhouse D. M., Calzone K. A., Mele C., Brenner D. E. (1993). Adherence to oral tamoxifen: a comparison of patient self-report, pill counts, and microelectronic monitoring. *Journal of Clinical Oncology*.

[24] De Geest S., Borgermans L., Gemoets H. (1995). Incidence, determinants, and consequences of subclinical noncompliance with immunosuppressive therapy in renal transplant recipients. *Transplantation*.

[25] Goodman R. (1997). The strengths and difficulties questionnaire: a research note. *Journal of Child Psychology and Psychiatry and Allied Disciplines*.

